# Stimulation of ovarian stem cells by follicle stimulating hormone and basic fibroblast growth factor during cortical tissue culture

**DOI:** 10.1186/1757-2215-6-20

**Published:** 2013-04-01

**Authors:** Seema Parte, Deepa Bhartiya, Dhananjay D Manjramkar, Anahita Chauhan, Amita Joshi

**Affiliations:** 1 Stem Cell Biology Department, National Institute for Research in Reproductive Health (NIRRH), JM Street, Parel, Mumbai, 400 012, India; 2 Experimental Animal Facility, NIRRH, JM Street, Parel, Mumbai, 400 012, India; 3 Department of Obstetrics & Gynecology, Seth GS Medical College and King Edward Memorial Hospital, A Donde Marg, Parel, Mumbai, 400 012, India; 4 Department of Surgical Pathology, King Edward Memorial Hospital, A Donde Marg, Parel, Mumbai, 400 012, India

**Keywords:** Primordial follicles, Organ culture, Germ cells, Ovarian stem cells, VSELs

## Abstract

**Background:**

Cryopreserved ovarian cortical tissue acts as a source of primordial follicles (PF) which can either be auto-transplanted or cultured *in vitro* to obtain mature oocytes. This offers a good opportunity to attain biological parenthood to individuals with gonadal insufficiency including cancer survivors. However, role of various intra- and extra-ovarian factors during PF growth initiation still remain poorly understood. Ovarian biology has assumed a different dimension due to emerging data on presence of pluripotent very small embryonic-like stem cells (VSELs) and ovarian germ stem cells (OGSCs) in ovary surface epithelium (OSE) and the concept of postnatal oogenesis. The present study was undertaken to decipher effect of follicle stimulating hormone (FSH) and basic fibroblast growth factor (bFGF) on the growth initiation of PF during organ culture with a focus on ovarian stem cells.

**Methods:**

Serum-free cultures of marmoset (n=3) and human (young and peri-menopausal) ovarian cortical tissue pieces were established. Cortical tissue pieces stimulated with FSH (0.5 IU/ml) or bFGF (100 ng/ml) were collected on Day 3 for histological and molecular studies. Gene transcripts specific for pluripotency (Oct-4A, Nanog), early germ cells (Oct-4, c-Kit, Vasa) and to reflect PF growth initiation (oocyte-specific Gdf-9 and Lhx8, and granulosa cells specific Amh) were studied by q-RTPCR.

**Results:**

A prominent proliferation of OSE (which harbors stem cells) and transition of PF to primary follicles was observed after FSH and bFGF treatment. Ovarian stem cells were found to be released on the culture inserts and retained the potential to spontaneously differentiate into oocyte-like structures in extended cultures. q-RTPCR analysis revealed an increased expression of gene transcripts specific for VSELs, OGSCs and early germ cells suggestive of follicular transition.

**Conclusion:**

The present study shows that both FSH and bFGF stimulate stem cells present in OSE and also lead to PF growth initiation. Thus besides being a source of PF, cryopreserved ovarian cortical tissue could also be a source of stem cells which retain the ability to spontaneously differentiate into oocyte-like structures *in vitro*. Results provide a paradigm shift in the basic understanding of FSH action and also offer a new perspective to the field of oncofertility research.

## Background

Culture of ovarian cortical tissue is carried out to allow the transition of resting phase primordial follicles (PF) to primary and secondary follicular stage. This is an interesting *in vitro* system to investigate the basic mechanisms involved in the activation and development of PF. This approach has also been implicated as a possible option to achieve biological parenthood in women cancer survivors and cryopreservation of ovarian cortical tissue is currently performed prior to cancer treatment for preservation of their fertility [1-4]. Currently, the practiced strategy involves auto-transplantation of cryopreserved ovarian tissue post cancer treatment. But, this may pose a high risk of re-introducing cancer in spite of remission from disease. Hence in the context of fertility preservation, *in vitro* maturation of PF from cryopreserved tissue is an excellent option for such females and also for those who wish to delay pregnancy for career or other reasons.

Ovarian cortical tissue culture however, cannot support follicle development of all stages, as the concentration and optimal timing of nutrient/growth factor requirements is stage-specific and not yet deciphered completely. Hence, a two-step procedure for follicular maturation has been proposed. The first stage involves serum-free culture of cortical tissue for 3-6 days, wherein the PF transitions to primary and secondary stage. This would be followed by *in vitro* maturation of cumulus-oocyte-complexes [2,3]. Recently, 3D culture of follicles for maturation of the oocytes has also shown promising results [5].

Till date, many intra- and extra-ovarian factors required for PF activation and follicle development in organ cultures have been studied. Role of follicle stimulating hormone (FSH), an extra-ovarian factor, during PF transition remains controversial. It has emerged as a survival factor [6] however the mechanisms underlying FSH action are not clear. PF growth is considered to be independent of FSH action as they (PF) lack FSH receptors [7,8]. Silva et al [9] reported that PF get activated spontaneously in caprine ovarian tissue culture and do not require FSH or EGF. Although both FSH and EGF stimulated an increase in oocyte and follicle size in intermediate and primary follicles in 5 days culture, no effect on proliferation or viability of follicles was observed in response to the treatment. Recently it has been suggested that FSH might act indirectly on the PF through paracrine factors secreted by large follicles or stromal cells [10]. This argument however remains unclear since FSH acts as a survival factor despite absence of large follicles in the cortical tissue pieces. Thus, more careful studies are necessary to understand the role of FSH as a survival factor.

Another important factor attributed to PF transition is basic fibroblast growth factor (bFGF). It is expressed in the ooplasm of primordial and primary follicles and also in the ovarian surface epithelium (OSE), smooth muscle cells surrounding blood vessels and corpus luteum [11]. Basic FGF when combined with FSH promoted PF survival and development in goats [12]. bFGF is also speculated to have a role in follicle recruitment and growth by stimulating granulosa cell proliferation [11]. Quennell et al [13] demonstrated the presence of bFGF transcripts in PF as well as decrease in its levels as the follicles grew in size. Garor et al [14] demonstrated the role of bFGF in human PF transition to primary follicles. Tang et al [15] reported that bFGF improved the effect of FSH on PF development and survival during long term culture of bovine ovarian tissue.

It was interesting to observe that FSH receptors and bFGF are expressed in the ovary surface epithelium [11,16] which also lodges ovarian stem cells [17-20]. Further bFGF is known to be a crucial component of culture medium required to maintain the pluripotency of human embryonic stem cells [21-24]. This emerging understanding motivated us to undertake the present study to investigate the *in vitro* effect of FSH and bFGF on PF growth with a focus on ovarian stem cells in the ovarian cortical tissues of marmoset and human ovaries. Day 3 (D3) was chosen as the time point for various analyses in the present study, since available studies suggest that spontaneous activation of PF occurs within 2-3 days *in vitro*[25-28]. Histological and q-RTPCR analysis have been carried out for various stem cell, oocyte and granulosa cell specific markers (please refer to the Additional file 1: Table S1) to study the effect of FSH and bFGF on ovarian stem cells and PF growth *in vitro*.

## Material and methods

### Procurement of ovarian tissue

The study for the use of marmoset ovaries was approved by the Institute Animal Ethics Committee of National Institute for Research in Reproductive Health (NIRRH). Use of human ovarian tissue for research was approved by the Human Ethics Committee of NIRRH and King Edward Memorial (KEM) Hospital. Ovarian tissue was collected from marmosets (n=3; normal cycling young adults, 3 to 4 years of age) and women (n=2; one young ovarian sample from a postmortem case of a 13 years old girl collected 6-7 hrs after death and one adult ovary from a peri-menopausal woman undergoing total abdominal hysterectomy) for the study. The young ovarian sample was collected at the time of autopsy from the Surgical Pathology unit and the peri-menopausal ovarian sample was procured from the Obstetrics and Gynecology unit, KEM Hospital. All the culture studies and further analysis were carried out at NIRRH.

### Ovarian cortical tissue culture

Ovarian tissue was transported in 0.9% normal saline-containing antibiotics (penicillin 100 U/ml, streptomycin 100 μg/ml; Invitrogen, Carlsbad, CA, USA) at ambient temperature to the laboratory. It was gently rinsed several times in calcium and magnesium-free Dulbecco’s phosphate-buffered saline (DPBS; Invitrogen) containing antibiotics. Ovarian cortical tissue (6-8 pieces of 1 mm^3^ size) were placed on 0.4 μm Millicell-CM inserts (Millipore, Bedford, MA, USA) fitted within six-well plates (NUNC, USA) and cultured at the interface between air and 1.1 ml of culture media, similar to that described previously [14]. The basal culture medium comprised of alpha-MEM (Invitrogen) containing 0.47 M ribonucleotides, 2 mM/L sodium pyruvate (Sigma-Aldrich, USA), 2 mM/L L-glutamine (Sigma-Aldrich), 1% insulin, transferrin, and selenium (Sigma-Aldrich), 0.05% Penicillin-Streptomycin (Invitrogen) and 2% human serum albumin (Sigma Aldrich). Ovarian tissues were treated with 0.5 IU/ml human urinary FSH [Utrofol, Kuanart Pharmaceuticals, Mumbai] and 100 ng/ml basic FGF [R & D Systems Inc, MN, USA]. Thus three different group of cultures were established including (A) untreated control (basal medium) (B) FSH treated [basal media supplemented with FSH], and (C) bFGF treated [basal media supplemented with bFGF]. Cultures were maintained in 5% CO_2_ at 37°C and were monitored regularly under an inverted microscope with Hoffman optics (Eclipse TE2000-S; Nikon, Japan). The cortical tissue pieces were collected from the cell culture inserts after three days and processed for histology and RNA extraction. The peri-menopausal ovarian tissue was treated only with FSH due to sample limitations. Details of various studies undertaken are shown in Table 1. In addition, cells observed to be released from the cortical tissue on to the surface of the cell culture inserts, were further cultured and regularly monitored up to three weeks.

**Table 1:** Experiments performed on marmoset and human ovarian cortical tissue

**Experiments undertaken**	**Details of ovary samples used in the study**					
	**Marmosets 1-3**		**Young human**		**Peri-menopausal human**	
***Organ culture***	**D3**		**D3**		**D3**	
FSH group	√		√		√	
bFGF group	√		√		ND	
***Histology***	**D3**		**D3**		**D3**	
FSH group	√		√		√	
bFGF group	√		√		ND	
***q-RTPCR***	**FSH**	**bFGF**	**FSH**	**bFGF**	**FSH**	**bFGF**
Oct-4A	√	√	√	√	√	ND
Nanog	√	√	√	√	√	ND
Oct-4	√	√	√	√	√	ND
c-Kit	√	√	√	√	√	ND
Vasa	√	√	√	√	√	ND
Gdf-9	√	√	√	√	ND	ND
Lhx8	√	√	√	√	ND	ND
Amh	√	√	√	√	ND	ND

√: experiment done; ND: experiment not done.

### Histology of ovarian cortical tissue

Cortical tissue pieces collected were fixed in 10% neutral buffered formalin for 12 hours at 4°C and subsequently dehydrated in a series of alcohol grades and processed for paraffin embedding using standard protocols. 5 μm thin sections were cut, stained with H & E and images of representative areas were captured using Nikon 90i Microscope (Nikon, Tokyo, Japan).

### RNA extraction and q-RTPCR studies

Levels of transcripts for pluripotent stem cells (Oct-4A, Nanog), early germ cells/oocyte specific (Oct-4, c-Kit, Vasa) and to reflect PF transition into primary follicles (oocyte-specific Gdf-9 and Lhx8, and granulosa cell specific Amh) were analyzed by q-RTPCR. Both total Oct-4 (comprising various isoforms) and Oct-4A (transcript specific for pluripotency) were studied to arrive at meaningful data showing the presence of VSELs and differentiated OGSCs respectively, as reported earlier [19].

Total RNA was extracted from the ovarian tissue pieces collected on D3 using standard TRIZOL (Invitrogen) method. RNA extraction method and cDNA preparation procedures were similar to those described earlier [19]. Real-time PCR analysis was performed using 2.5 μL of cDNA and 10 pmol of gene specific primers (Table 2). The comparative threshold cycle (Ct) values were calculated using CFX 96 Real-Time PCR system (Bio-Rad Laboratories, CA, USA) using SYBR Green chemistry (Bio-Rad).

**Table 2:** List of primer details and PCR cycling conditions used in the study

**Gene**	**Primer sequence**	**Annealing temperature (°C)**	**Amplicon size (bp)**
***Pluripotent markers (Human)***			
**Oct-4A**	***F*:** AGCCCTCATTTCACCAGGCC	57°C	448
	***R*:** TGGGACTCCTCCGGGTTTTG		
**Nanog**	***F*:** TGCAAATGTCTTCTGCTGAGAT	57°C	285
	***R*:** GTTCAGGATGTTGGAGAGTTC		
***Early germ cell markers (Human)***			
**Oct-4**	***F*:** GAAGGTATTCAGCCAAACGAC	55°C	315
	***R*:** GTTACAGAACCACACTCGGA		
**Oct-4 (marmoset)**	***F*:** CCCCTGGTGCTGTGAAGCTGG	64°C	124
	***R*:** CCCCAGGGTGAGCCCCACAT		
**c-Kit**	***F*:** CCTGGGATTTTCTCTGCGTT	60°C	376
	***R*:** ATTGGTCACTTCTGGGTCTG		
**Vasa**	***F*:** GAC TGC GGC TTT TCT CCT ACC	55°C	418
	***R*:** TTT GGC GCT GTT CCT TTG AT		
***PF transition markers (Human)***			
**Amh**	***F*:** CACCTGGAGGAAGTGACCTG	60°C	202
	***R*:** CCACCGCTAACACCAGGTAG		
**Amh (marmoset)**	***F*:** ACCTGGAGGAAGTGACATGG	64°C	190
	***R*:** ACCAGGTAGTGGGTGTCTCG		
**Gdf-9**	***F*:** CTCCTGGAGACCAGGTAACAGGAAT	65°C	291
	***R*:** TGCACACACATTTGACAGCAGAGG		
**Lhx8**	***F*:** CAAGCACAATTTGCTCAGGA	62°C	230
	***R*:** GGCACGTAGGCAGAATAAGC		
***Housekeeping gene (Human)***			
**18S**	***F*:** GGAGAGGGAGCCTGAGAAAC	60°C	171
	***R*:** CCTCCAATGGATCCTCGTTA		

Amh (granulosa cells), Gdf-9 and Lhx8 (oocyte specific).

Amplification conditions comprised of an initial denaturation at 95°C for 3 min, followed by 45 cycles of denaturation at 94°C for 30s, annealing for 30s at respective temperature followed by elongation at 72°C for 50 sec. The melt curve analysis was performed at the end of 45 cycles to determine the homogeneity of the amplified products. The Ct values generated by CFX manager software (Bio-Rad) were normalized with respect to housekeeping gene 18S rRNA for all samples, and the Delta-Delta Ct values for each gene transcript were computed manually by subtracting delta Ct value of treated sample with that of its respective untreated control. Data obtained by q-RTPCR analysis using three adult marmosets, one each of young and peri-menopausal human ovarian samples are represented individually. All reactions were carried out in duplicates and the experiments were repeated thrice to determine the reproducibility of the results.

## Results

### Microscopic examination of ovarian cortical tissue

**Figure 1:**
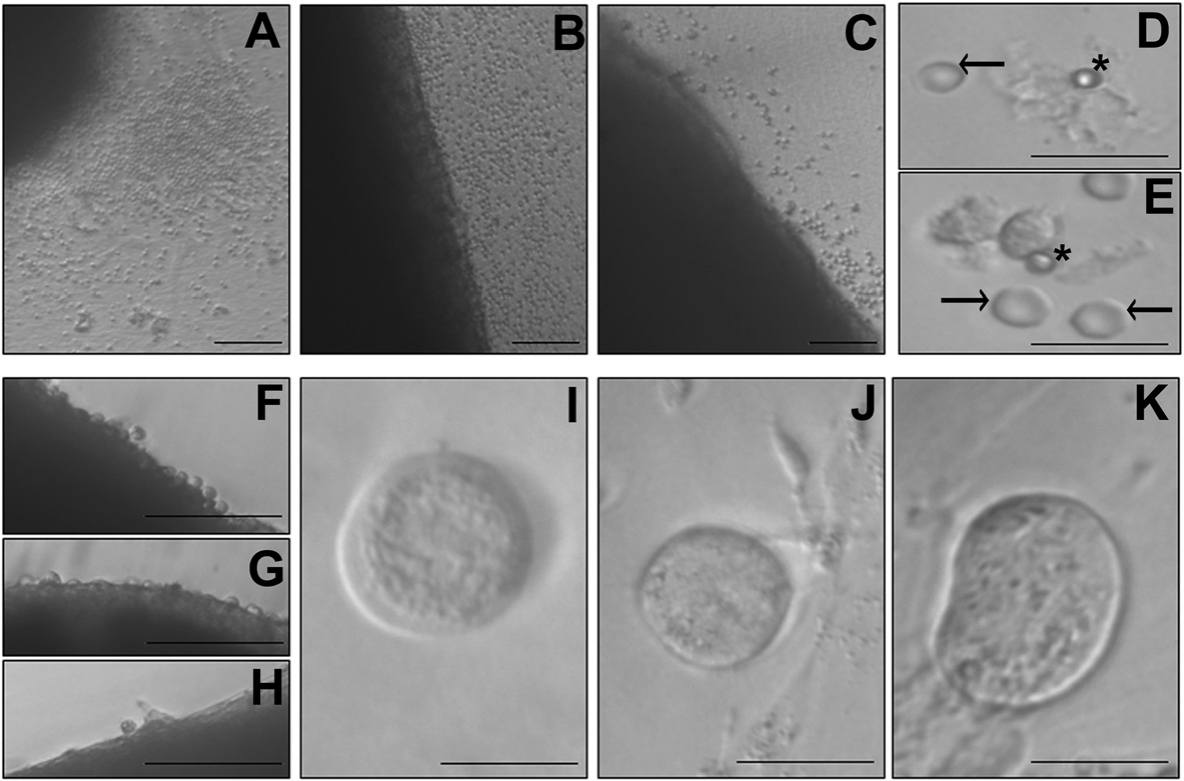
**Microscopic observation of ovarian cortical tissue:** A large number of cells were observed on the membrane of cell culture inserts in (**A**) marmoset, (**B**) young and (**C**) peri-menopausal human ovary. (**D** &**E**) Stem cells (asterix) which exist in the OSE layer were visualized at higher magnification on the inserts along with RBCs (arrow) and epithelial cells. (**F**-**H**) In peri-menopausal ovarian tissue, these cells appeared to be released from the ovarian surface in the presence of (**F**) FSH and (**G**) bFGF compared to the (**H**) untreated control. The released cells were further cultured for three weeks and spontaneously differentiated into oocyte-like structures in (**I**) marmoset (**J**) young and (**K**) peri-menopausal human ovary samples. Scale bar in **A**-**C**, **F**-**H** = 20 μm and **D**, **E**, **I**-**K** = 10 μm.

Cortical tissue pieces cultured on the membrane of cell culture inserts appeared dark under the inverted microscope (Figure 1A-C, F-H). However, few cells including RBCs interspersed with VSELs & OGSCs (ranging in size from 1-7 μm) and larger epithelial cells (15-20 μm) were observed to be released onto the culture insert membrane surface in both marmoset (Figure 1D) and human ovarian tissue culture (Figure 1E). OSE layer at high magnification was visible in human cortical tissue (Figure 1 F-H).

Regular monitoring of the released cells on the cell culture inserts showed that the epithelial cells attached to membrane surface and formed a monolayer. The stem cells (VSELs and OGSCs) appeared to grow in size and differentiate spontaneously into oocyte-like structures (Figure 1I-K) by the end of three weeks similar to ovary surface epithelium cultures reported earlier by various groups [18,19,29].

### Histological analysis of ovarian cortical tissue

#### Marmoset

**Figure 2:**
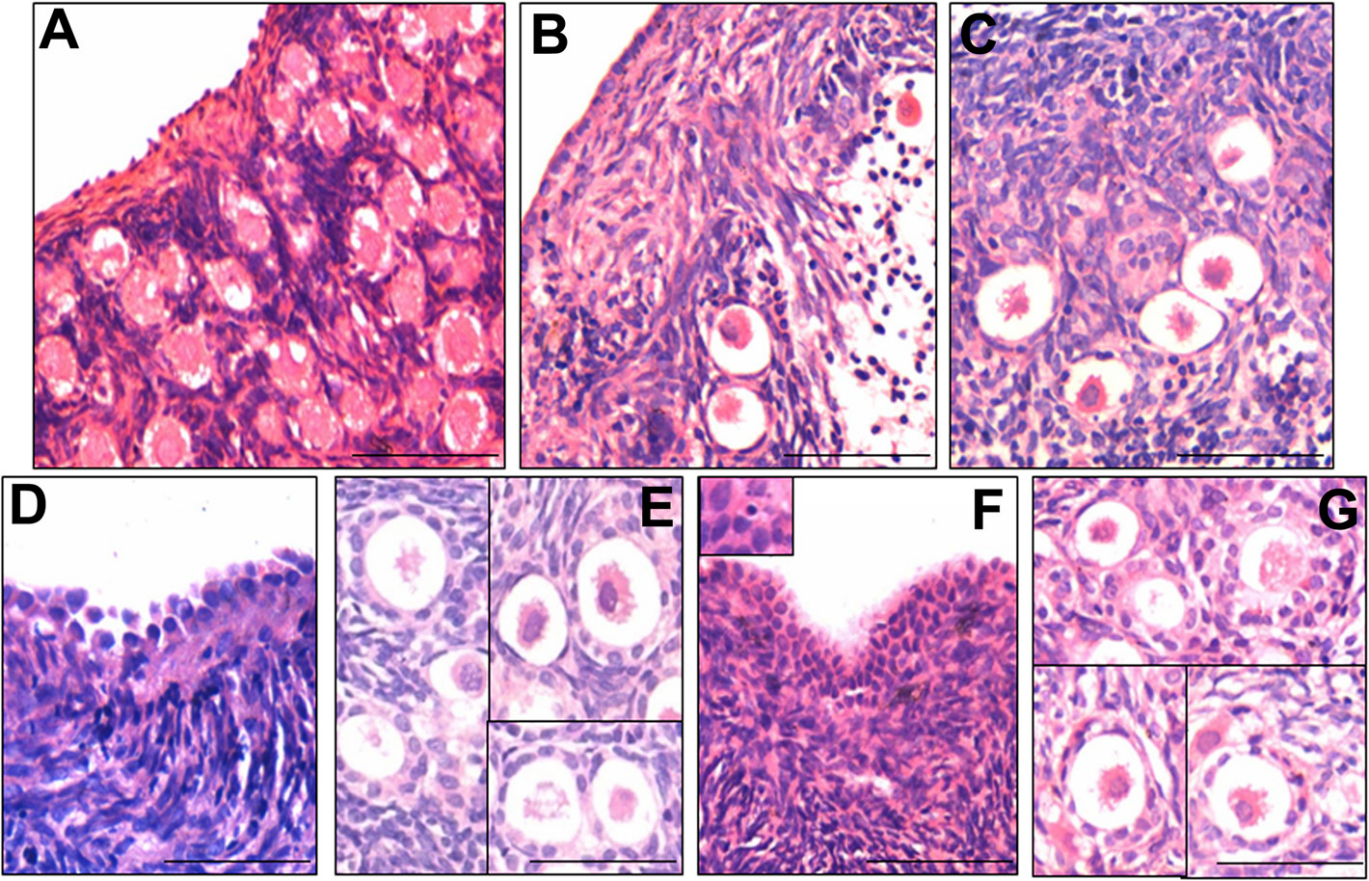
**Histology of marmoset ovarian cortical tissue:** (**A**) OSE and PF were visualized in cortical tissue sections after Haematoxylin & Eosin staining prior to culture. (**B** &**C**) Three days culture in untreated control showed minimal OSE, few PF with pycnotic nuclei and disorganized cortex (**D** &**E**) Ovarian tissue sections after FSH treatment showed OSE proliferation and PF growth and transition to primary and secondary stage as evident by change in shape of the surrounding granulosa cells from flat and single layered in untreated control (**A**) to cuboidal and 2-3 layers after growth (**E**). (**F** &**G**) Ovarian tissue sections after bFGF treatment showed OSE proliferation and PF growth and transition to primary and secondary stage. Inset in F shows a darkly stained stem cell amongst OSE cells. Scale bar = 20 μm.

Sections of the ovarian cortical tissue prior to culture demonstrated intact OSE layer and several intact PF (Figure 2). The OSE comprised of discontinuous and small cuboidal cells. Large numbers of PF were observed with a single layer of flattened granulosa cells (GC) surrounding the central oocyte. No signs of pycnotic nuclei, eosinophilia of ooplasm or clumping of the chromatin material were detected in the PF (Figure 2A), suggesting that the follicles were healthy.

The untreated control ovarian tissue on D3 in culture exhibited disorganization of the cortical stroma. The OSE layer appeared intact and continuous comprising of short cuboidal cells whereas the PF showed no initiation of growth (Figure 2B, C). The tissue sections of FSH treated cortical tissue revealed relatively better preserved histology. The OSE appeared multilayered, exhibited hypertrophy and comprised of tall and columnar epithelial cells (Figure 2D). The granulosa cells in few follicles appeared cuboidal rather than flat, suggesting PF growth. Primary follicles with few intact PF were also observed (Figure 2E). The tissue harvested at D3 post treatment with bFGF appeared intact, similar to the FSH treated group. OSE appeared multi-layered with small spherical cells having high nucleocytoplasmic ratio and Hematoxylin stained dark nuclei were visible in some fields (inset, Figure 2F), which resembled the VSELs. Few PF, primary and secondary follicles were observed in the cortex (Figure 2G).

#### Human

**Figure 3:**
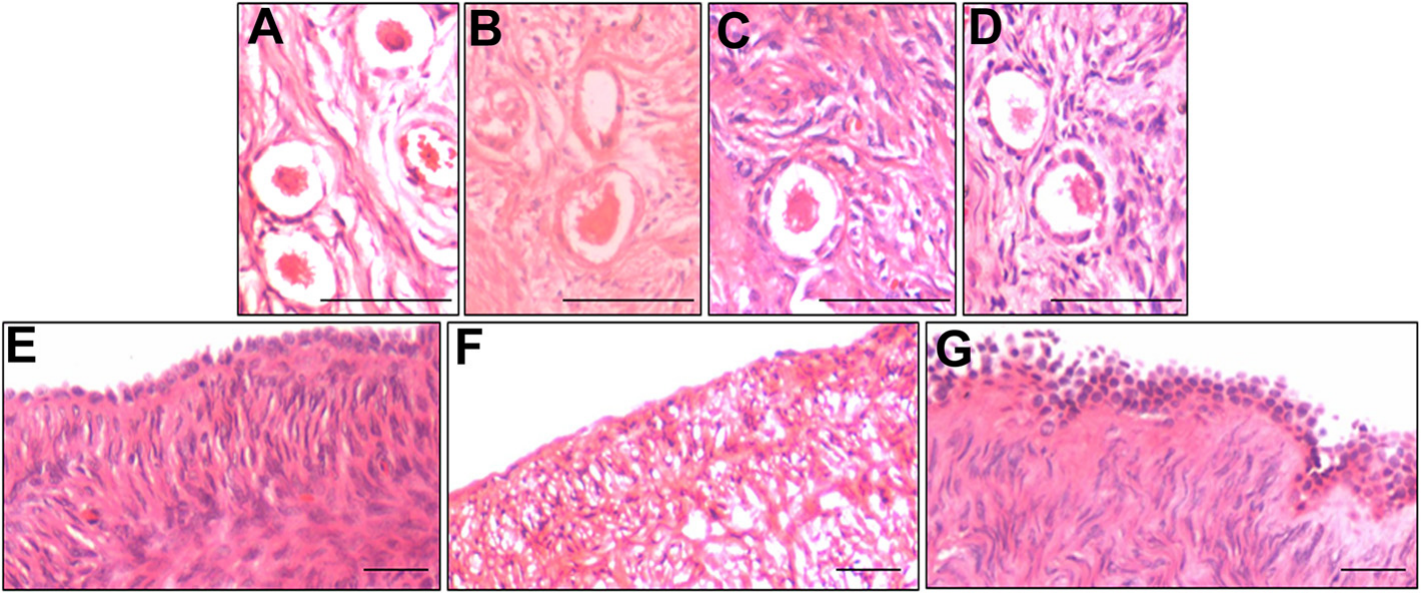
**Histology of human ovarian cortical tissue.** (**A**-**D**) Young ovarian tissue sections and (**E**- **G**) Peri-menopausal ovarian tissue sections (**A**) OSE was not visualized but PF were seen in the cortex prior to culture (**B**) Untreated group revealed disorganized stroma with distorted PF. Healthy primary follicles were observed in the cortex after three days culture with (**C**) FSH and (**D**) bFGF treatment. (**E**) Prominent single layer of OSE and stroma devoid of follicles in peri-menopausal ovarian tissue (**F**) Loss of epithelial cells and disorganized stroma were evident after three days culture without any treatment (**G**) FSH treatment resulted in proliferation and multi-layered appearance of OSE. Scale bar = 20 μm.

In case of young human ovarian tissue, the OSE was discontinuous (data not shown) and relatively few intact PF were observed in the cortex before initiation of culture (Figure 3A). Some degree of transition of PF with flat GC to primary stage with cuboidal GC was observed in both FSH and bFGF treated groups, compared to untreated control sample which comprised majorly of PF (Figure 3B-D). The peri-menopausal ovarian sample had a well-defined OSE but was devoid of any follicles (Figure 3E). The OSE was unaffected after three days culture in the untreated group (Figure 3F), however it was multilayered and prominent after FSH treatment (Figure 3G).

### Expression of pluripotent stem cells, germ cells and PF transition specific transcripts

Levels of transcripts for pluripotent stem cells (Oct-4A, Nanog), early germ cells/oocyte specific (Oct-4, c-Kit, Vasa) and to reflect PF transition into primary follicles (oocyte-specific Gdf-9 and Lhx8 and granulosa cell specific Amh) were analyzed. The transition specific markers were not studied in peri-menopausal tissue as this sample lacked PF. There was considerable biological variation in q-RTPCR results between samples but the basic trend remained similar while comparing data obtained in marmosets with that from human samples. Results were represented as fold change values over untreated control taken as 1 after normalizing with housekeeping gene (18S rRNA gene) and graphs were plotted on a logarithmic scale.

#### Pluripotent stem cell markers

**Figure 4:**
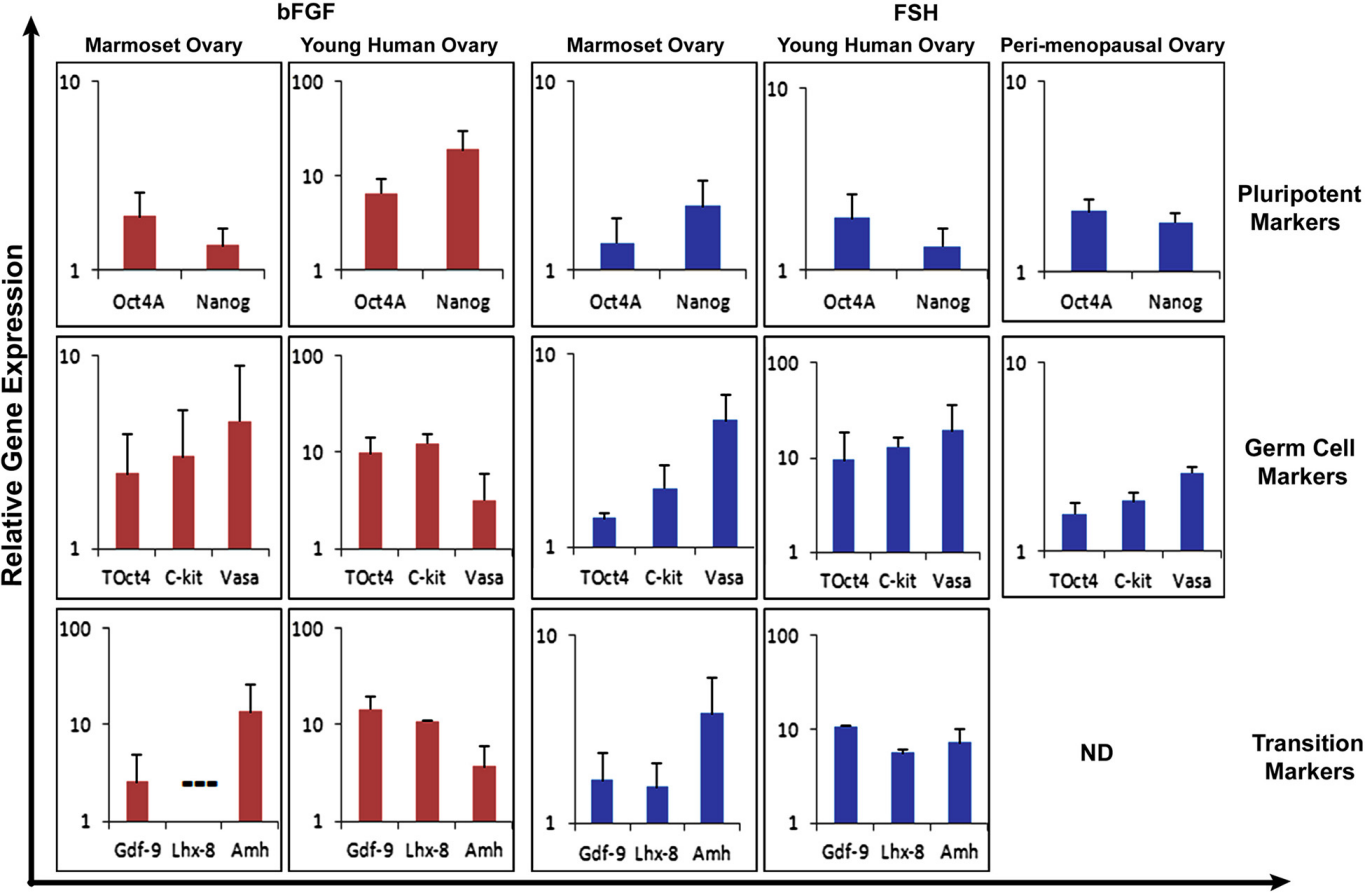
**Effect of bFGF and FSH on expression of pluripotent, germ cell and PF transition specific markers by q-RTPCR:** Expression of pluripotent markers (Oct-4A and Nanog), early germ cell markers (Oct-4, c-Kit and Vasa) and PF growth initiation markers (oocyte specific Gdf-9, Lhx8 and granulosa cells specific Amh) were clearly up-regulated in response to bFGF and FSH treatment compared to untreated control (except Lhx-8 in marmoset after bFGF treatment). Values depicted are mean fold change in logarithmic scale obtained with respect to untreated controls taken as 1 and 18S rRNA as house-keeping gene. Marmoset data represents results obtained from three animals, whereas human data is representative of one young and one peri-menopausal ovary. *ND:Experiment not done*.

Both Oct-4A and Nanog transcripts showed increased expression in marmoset and human cortical tissues treated with FSH and bFGF (Figure 4). Typically human sample showed an increase in Oct-4A transcripts in response to FSH treatment whereas Nanog mRNA was expressed to higher extent in bFGF treated group. FSH treatment in marmoset ovarian tissue showed higher expression of Nanog whereas bFGF treated group showed higher expression of Oct-4A transcripts. Typically young human ovary samples exhibited high fold change values for Nanog in both treated groups, whereas expression of both transcripts was comparable in the adult (peri-menopausal) sample.

#### Early germ cell markers

A prominent and uniform up-regulation of germ cell specific markers was noticed after FSH and bFGF treatment in both marmoset and human samples compared to untreated control. Of all the three markers studied, Vasa showed highest fold change followed by c-Kit and Oct-4 in all the samples except young human sample treated with bFGF. Expression of early germ cell markers was most pronounced in young human sample. Although expression of c-Kit increased minimally with FSH treatment, the bFGF group did not exhibit any fluctuation in fold change value in marmoset sample. There was a consistent up-regulation of c-Kit in human samples in both treatment groups (Figure 4).

#### Markers to study PF growth and transition to primary follicle

An increase in the expression of transition specific markers was noted in all the groups suggesting PF growth initiation (Figure 4).

## Discussion

The present study was undertaken to evaluate the effect of FSH and bFGF on PF transition from resting to growing stage during marmoset and human ovarian cortical tissue culture. Results revealed that in addition to the transition of resting PF to growing phase (as indicated by histological studies and increased expression of Gdf-9, Lhx-8 and Amh transcripts by q-RTPCR), proliferation of the OSE cells along with a release of stem cells onto the cell culture inserts was observed in response to FSH and bFGF. Also an increased expression of pluripotent stem cell (Oct-4A, Nanog) and germ cell (Oct-4, c-Kit, Vasa) specific markers was observed. A higher fold increase of stem cell markers was observed by q-RTPCR in the young human ovarian sample. Stem cells released on the inserts retained the ability to spontaneously differentiate into oocyte-like structures as reported earlier by our group [19]. Results of the present study demonstrate a direct role of FSH and bFGF on ovarian stem cells and PF growth initiation. These observations challenge the existing paradigm which suggests that FSH exerts indirect action on the germ cells via its receptors on granulosa cells and that initial follicular growth is gonadotropin independent [30].

Both FSH and bFGF induced increase in proliferation of OSE and also increased expression of pluripotent markers Oct-4A and Nanog in all the groups including the peri-menopausal human ovarian sample devoid of PF. Expression of pluripotent stem cell markers Oct-4A and Nanog indirectly suggested the presence of pluripotent very small embryonic-like stem cells (VSELs) in the OSE as reported earlier by our group [19]. VSELs appear to increase in number after FSH or bFGF treatment. The increased expression of Oct-4A and Nanog as revealed by q-RTPCR, however may not reflect the true increase as large numbers of these stem cells were shed on to the membrane of cell culture insert, which were not processed for RNA extraction. Presence of stem cells in peri-menopausal ovary and their increase after FSH treatment suggests that stem cells persist and retain the ability to proliferate but menopause sets in due to a compromised microenvironment (somatic niche) which does not allow the stem cells to differentiate and assemble into PF as suggested earlier by our group [19,20,31] and also others [32].

A similar increase in pluripotent stem cells and PF assembly in FSH analog treated mice has been reported by us [31]. It was intriguing to observe that FSH exerts action on the cortical tissue pieces comprising mainly of PF which are reported to be devoid of FSH receptors [8]. FSH action leading to follicular maturation is mediated via the granulosa cells of growing follicles which express FSH receptors [30,33]. Further studies are required to investigate the mechanism of FSH action on the ovarian stem cells.

Two distinct stem cell populations exist in the OSE including VSELs and their immediate descendants OGSCs [19]. We have earlier proposed that in the mammalian ovary, VSELs give rise to OGSCs just like the VSELs in the testis give rise to A_dark_ spermatogonial stem cells [19,20]. These immediate descendant ‘progenitors’ have cytoplasmic OCT-4B and divide rapidly whereas the VSELs express nuclear OCT-4A and are relatively quiescent. Presence of these two populations of stem cells in the gonads is in agreement with the recent reports demonstrating two distinct stem cell populations in various body tissues [34,35].

Available literature suggests that transition of PF into primary to secondary follicles occurs within 8-20 days of ovarian cortical tissue culture. We were indeed surprised to observe varying degree of damage to the PF in the histological sections. In view of this, survival of PF in extended cultures is rather intriguing. The poor morphology could be due to compromised culture condition or a fixation artifact in the present study. But we used culture conditions based on recent studies [14] and standard fixation protocols. This forced us to contemplate whether it is the pre-existing PF in cortical tissue or new follicles assembled from the ovarian stem cells which further grow into primary to secondary stage in the long-term (conducive environment of) culture conditions. We have earlier proposed that PF assemble in the OSE wherein the VSELs differentiate into oocytes and the epithelial cells undergo epithelial-mesenchymal transition to give rise to granulosa cells [19,20,31]. Garor et al [14] also observed an increased number of PF in bFGF treated ovarian cortical tissue culture (reported 301 follicles in the presence of 50 ng/ml of bFGF compared to 151 follicles in untreated control; presumably from similar number of cortical tissue pieces). However, it remains unclear whether the increase in number of follicles reported by them was due to better survival or *de novo* PF assembly from the stem cells in response to the treatment. More studies need to be undertaken in this direction. In the present study, an increased expression of transition associated markers for oocyte (Gdf-9 and Lhx-8) and granulosa cells (Amh) were observed after the treatment, suggestive of PF transition into primary follicles, in agreement with published literature [36-38].

Stimulation of pluripotent stem and early germ cell specific markers by bFGF was not surprising since bFGF receptors are reported to be present in the OSE [11]. Expression of above and PF transition specific markers were increased by several folds more after bFGF treatment compared to FSH, especially in young human ovarian tissue. Our results are in agreement with Garor et al. [14] who have also reported enhanced PF development in response to bFGF treatment during ovarian cortical tissue culture. It is likely that FSH and bFGF interact under normal circumstances in the ovary, to facilitate neo-oogenesis followed by PF assembly in mammalian ovary. FSH may exert its action via bFGF, as direct bFGF treatment leads to the enhanced effect. Especially the young human ovarian tissue responded more prominently to treatment with bFGF (>8 to 9 folds higher expression in bFGF compared to >2-3 folds in case of FSH group). However, more samples need to be studied before arriving at a final conclusion. Synergistic effect of FSH and bFGF in driving stem and germ cell development also requires further investigation.

The young human ovarian sample procured from autopsy, used in the present study, showed presence of few PF whereas the stem cells survived post death and exhibited increased stimulation by FSH and bFGF treatment. A better survival of stem cells compared to the PF could be because stem cells survive hypoxic conditions better in agreement with a recent study wherein viable stem cells were isolated seventeen days post death and they retained the ability to differentiate into skeletal muscles [39].

To conclude, culture of ovarian cortical tissue pieces is an excellent tool to study ovarian stem cell biology along with PF transition. Both FSH and bFGF stimulate the ovarian stem cells and probably aid in the assembly of stem cells as PF during extended cultures. It will be of great interest to study whether long-term culture of peri-menopausal ovarian tissue can result in PF assembly from the stem cells in response to FSH and bFGF treatment. Results of the present study have translational potential for both oncotherapy and infertility.

## Competing interests

The authors declare that they have no competing interests.

## Authors’ contributions

SP carried out experimental work, data analysis, interpretation and manuscript drafting. DB was responsible for conceptualizing the contents of the research article, planning experiments, providing scientific inputs, data interpretation and critical drafting of manuscript. DM performed ovariectomy of marmoset samples and reviewed the manuscript. AC and AJ were responsible for providing human ovarian tissue samples and reviewing the manuscript. All authors read and approved the final manuscript.

## Supplementary Material

Additional file 1: Table S1 Details of markers used for characterization of pluripotent stem cells, germ cells and primordial follicle transition. Click here for file
