# Supplementary material for: Stimulation of ovarian stem cells by follicle stimulating hormone and basic fibroblast growth factor during cortical tissue culture
Source: J Ovarian Res. 2013 Apr 1;6:20. doi: 10.1186/1757-2215-6-20 (PMC3635909; doi:10.1186/1757-2215-6-20)
Supplement: Additional file 1: Table S1 — Details of markers used for characterization of pluripotent stem cells, germ cells and primordial follicle transition. [file 1757-2215-6-20-S1.doc]

**Supplement Table**

**Details of markers used for characterization of pluripotent stem cells, germ cells and primordial follicle transition**

| **SR NO** | **GENE** | **LOCALIZATION** | **FUNCTIONAL SIGNIFICANCE** | **MUTANT/NULL OUTCOME** | **REFERENCE** |
| --- | --- | --- | --- | --- | --- |
| 1 | Oct4A | Oct-4A isoform is nuclear in pluripotent stem cells | Oct4A is a homeodomain, octamer binding nuclear transcription factor of the POU family responsible for the stemness properties, critically involved in self-renewal of undifferentiated ES cells and also known to be expressed in VSELs.  It is normally found in the pluripotent stem cells of pre-gastrulation embryos, early cleavage-stage embryos, and the ICM of the blastocyst. It is expressed in undifferentiated pluripotent cells and tumors. | Oct-4 gene knockdown promotes differentiation, thereby implicating its role in human ES cell self-renewal. | Scholer *et al.* 1990,  Cauffman *et al*. 2006,  Lee *et al*. 2006, Bhartiya *et al*. 2010,  Parte *et al*. 2011 |
| 2 | Nanog | Nuclear  Expressed in the morula, ICM and EG cells, in the epiblast at 6 days and in PGCs of genital ridges between 11.5 and 12.5 days. It is expressed in germ cells of the fetus and in some germ cell tumors of the gonads. | Nanog is a transcription factor critically involved with pluripotency of ES cells and contains 1 homeobox DNA-binding domain.  Plays bi-functional role i.e. activates genes required for stem cell self-renewal and prevents differentiation towards extra-embryonic endoderm and trophectoderm lineages.  It also regulates the expression and activates functions of Oct-4 and Sox-2 genes to establish ES identity. | Nanog-deficient ES cells show loss of pluripotency.  Nanog-/- mice are embryonic lethal.  Conditional deletion of Nanog by TNAP-Cre, results in decreased number of the founder population of PGCs at E7.5, and remaining PGC die during migration. | Mitsui *et al.* 2003, Yamaguchi *et al.* 2009,  Jagarlamudi *et al.* 2012 |
| 3 | Oct4 | Oct-4B isoform localized in cytoplasm of progenitors viz. OGSCs and GCN | Oct-4 (octamer-binding transcription factor 4) also known as POU5F1 (POU domain, class 5, transcription factor 1).  Can form a heterodimer with Sox-2, to enable two proteins to bind DNA together.  It is a germ-line specific maternally expressed factor expressed by PGCs and germ cells during embryonic development. Also known to be expressed in tissue-specific progenitors and oocytes. | Mouse embryos that are deficient in Oct-4, or have low expression levels do not form ICM, lose their pluripotency and undergo differentiation into tropho-ectoderm  Germ cell specific deletion of Pou5f1 using TNAP-Cre (tissue-non-specific alkaline phosphatase-Cre-recombinase) transgenic  mice at E7.5, leads to apoptosis of PGC around E10.5 | Scholer *et al.* 1990,  Kehler *et al.* 2004, Cauffman *et al*. 2006,  Lee *et al*. 2006,  Bhartiya *et al*. 2010,  Parte *et al*. 2011, Jagarlamudi *et al.* 2012 |
| 4 | c-Kit | Plasma membrane of PGC, Oocyte, Granulosa, Theca cells, surface epithelium and Ovarian tumor.  c-Kit was confined to the oocyte and granulosa cells in primary and secondary follicles by RT PCR.  Western blot analysis revealed presence of soluble c-kit protein in the follicular fluid. | C-KIT controls cellular processes, including migration, proliferation, differentiation and survival of follicles in long-term culture.  Autocrine/Paracrine roles of c-Kit/KL system during: PF assembly, maintenance of the PF reserve and in the primary to secondary transition and throughout folliculogenesis  Controls survival of human ovarian follicles during early follicular development. | Blocking c-Kit receptor induces follicular atresia in mice and human ovarian cultures. Mutation in c-Kit gene affects PGC survival and induces sterility in mice.  Neutralization of c-Kit at post natal day5 by injecting ACK2 antibody caused disturbances in initial follicle recruitment, PF activation, antrum formation and granulosa cell proliferation in rat ovary. | Buehr *et al*. 1993, Yoshida *et al*. 1997,  Tanikawa *et al*. 1998,  Parrot & Skinner, 1999,  Reynaud *et al*. 2000, Eppig, 2001,  Nilsson & Skinner 2001,  Carlsson *et al*. 2006, Hutt 2006 |
| 5 | Vasa | Plasma membrane and nucleus  PGC, Oocyte of  Primordial, Primary, Secondary, Antral,  Preovulatory | Vasa is a member of the DEAD-box protein family with evolutionarily conserved role in germ line across *C. elegans*, *Xenopus*, zebrafish, mouse, and rat.  VASA protein is present in fetal and adult gonadal germ cells in both males and females and most abundant in spermatocytes and mature oocytes.  It is involved in germline cyst development, oocyte differentiation, gurken and *oskar* mRNA translation and oocyte polarity in drosophila.  It has an essential role in development, specification, proliferation and maintenance of germ cells.  Animals with Vasa^+^ multipotent cells are capable of tissue regeneration in the adult to varying degrees.  VASA^+^ FGSC were isolated from mice ovaries by FACS | Vasa null female mice show normal development but are infertile, in male mice its deficiency affects germ cell proliferation and differentiation causing infertility.  Hereditary infertility syndromes mapping to the chromosomal region of *VASA* has not yet been found in humans. | Raz *et al.* 2000, Tanaka *et al.*2000,  Castrillon *et al.*2000,  Newmark *et al.*2008 |
| 6 | AMH | GC of growing follicles. It is detected in sheep & human GC at perinatal period. In case of human it is undetectable after menopause. In rat & mouse, AMH & its mRNA are absent in pre GC of PF, but present in primary follicle of GC.  AMH & AMH type II mRNA expression (in adult rat ovary) is lost from atretic follicles. | Anti-Mullerian Hormone belongs to TGF β superfamily.  AMH has the least inter- and intra-cycle variability, thus serves as a good hormonal marker for evaluation of ovarian reserve in primary ovarian insufficiency used at clinical settings in random blood samples.  It is an indicator of antral follicles in ovary and the number of oocytes retrieved.  AMH functions *d*uring initial recruitment-reduced activation and cyclic recruitment- reduced responsiveness of growing follicles  Growing follicles produce AMH which acts as negative regulator on neighboring PF & inhibits their recruitment. Negative regulation of PF activation is not known as PF lack AMH receptor .On contrary Schmidt et al 2005 showed +ve effect on PF recruitment, survival and growth | In AMH^-/-^ mice number of growing follicles increases.  In AMH^-/-^ mice the PF pool gets depleted, rate of depletion accelerates with advancing age.  Growing follicles in AMH ^-/-^ mice show increasing sensitivity to FSH & faster rate of recruitment. | van Rooij *et al.* 2002,  Schmidt *et al*. 2005,  Ebner *et al.* 2006, Adhikari *et al*. 2009 |
| 7 | GDF-9 | expressed in oocyte cytoplasm | Growth differentiation factor (GDF)-9 is a cystine knot-containing paracine hormone of the TGF-β superfamily produced by the oocyte.  GDF-9 controls granulosa cell growth and differentiation during early ovarian folliculogenesis and regulates cumulus cell function and ovulation rate in the later stages of this process.  It stimulates PF to 1F progression. | Follicle development is arrested at the 1F stage in GDF-9 null mice which leads to complete infertility. Although oocyte growth and zona pellucida formation may occur normally, but other aspects of oocyte differentiation are compromised. | Dong *et al*. 1996,  Vitt *et al*. 2000 |
| 8 | Lhx8 | expressed in nucleus of  oocytes and germ cells | Lim homeodomain (a zinc finger structure) germ cell specific transcriptional regulator downstream of Sohlh1 and critical in fertility, also involved in development of cholinergic neurons in mouse forebrain and mesenchymal cells.  Lhx8 is a critical factor essential for maintenance and differentiation of the oocyte during early oogenesis.  Also brings about down-regulation of the Nobox pathway. Expression increases during PF activation.  *Roles:* DNA binding protein, transcription factor, cell development/differentiation and patterning of various tissue types, forebrain neuron development, female gonad development, odontogenesis of dentine-containing teeth. | LHX8(-) mice develop a cleft secondary palate due to failure of palatal shelves to connect and fuse properly.  Lhx8(-/-) ovaries fail to maintain the PF as transition to growing follicular stage does not occur.  Lhx8(-/-) ovaries mis-express oocyte-specific genes such as Gdf9, Pou5f1 and Nobox.  Also a down-regulation of Kit and Kitl in Lhx8(-/-) ovaries is observed which causes oocyte loss. | Pangas *et al*. 2006,  Choi *et al*. 2008, Zhang *et al*. 2012 |
| 9 | bFGF | Oocyte of PF, growing follicles and GC | Basic fibroblast growth factor (b-FGF/FGF-2) is (18 kDa protein) part of 19-member family of heparin-binding growth factor.  Is a signaling molecule for various developmental, physiological and pathological functions, acts in cell differentiation, migration and angiogenesis in many tissues.  *In vitro* it promotes growth of PF and 1F by increasing the KL mRNA, proliferation of granulosa and theca cells, suppressor of PCD in GC. Acts in synergism with SCF or insulin to stimulate 1F & 2F growth in rat. Responsible for PF development and survival in goat when coupled with FSH.  bFGF may mediate follicular activation through enhancement of KL expression. | FGF-2^-/-^ mice are viable and fertile, subtle fertility defects if any not studied or if other GF compensate lack of FGF-2 *in vivo* not known.  Neutralizing with antibody against SCF receptor and AMH abolished the positive effect of bFGF | Shikone *et al*. 1992,  van Wezel *et al*. 1995,  Wandji *et al.* 1996,  Kezele *et al.* 2002,  Ben Haroush *et al.* 2005,  Nilsson *et al*. 2001, 2004, 2007,  Matos *et al.* 2007a,  Matos *et al.* 2007b,  Garor *et al*. 2009, Matos *et al*. 2011 |
| 10 | FSH | Anterior pituitary  Its receptors are present on GC of primary follicles and oocytes | Follicle stimulating Hormone (FSH) is a hetero dimeric (35.5 kDa) glycoprotein hormone which acts by binding to its receptor expressed on GC and recently in oocytes, suggesting additional sites of action in ovary.  FSH promotes GC proliferation via paracrine factors such as IGF-1 and activin, regulates expression of KL, GDF-9 and BMP-15 in murine follicles and is implicated in PF activation  *FSH stimulates the expression of:*  FGF-2 receptors in GC and enhances its stimulatory effect on 1F growth until 14 days of organ culture.  Stimulates antrum formation and steroidogenesis in granulosa cells  Increase in follicular diameter and proliferation of granulosa cells  FSH may also stimulate the stem cells lodged in the OSE through FSHR3 receptor based on the study carried out on PMSG treated mice – thereby augment neo-oogenesis and PF assembly | Female mice were infertile if FSHR is knocked out (KO) or its ligand (FSHβKO) is disrupted.  GC in FSH-deficient mice demonstrates increase in FSHR mRNA, and decreases in P450 aromatase, serum/gluco-corticoid-induced kinase, and inhibin/activin subunit mRNAs.  Follicles cultured without FSH showed signs of degeneration after 7 days organ culture and exhibited more clear degenerative features, like ooplasm vacuolization.  FSH receptor mutation in women cause arrest at PF stage in their ovaries. | Aittomaki et al., 1996, O’Shaughnessy *et al.* 1996,  Joyce *et al.* 1999, Burns *et al.* 2001, Meduri *et al.* 2002,  Abel *et al.* 2003, Thomas *et al.* 2005,  Van den Hurk & Zhao, 2005, Matos *et al*. 2007c & 2011,  Bhartiya et al, 2012b |

**References for Supplement Table**

1. Scholer HR, Ruppert S, Suzuki N, Chowdhury K Gruss P: **New type of POU domain in germ line-specific protein Oct-4.** *Nature* 1990, **344**:435-439.
2. Cauffman G, Liebaers I, Van Steirteghem A and Van de Velde H: **POU5F1 isoforms show different expression patterns in human embryonic stem cells and preimplantation embryos**. *Stem Cells* 2006 **24**:2685-2691.
3. Lee J, Kim HK, Rho JY, Han YM Kim J: **The human OCT-4 isoforms differ in their ability to confer self-renewal.** *J Biol Chem 2006,* **281** (44): 33554-33565.
4. Bhartiya D, Kasiviswanathan S, Unni SK, Pethe P, Dhabalia JV, Patwardhan S and Tongaonkar HB: **Newer insights into pre-meiotic development of germ cells in adult human testis using Oct-4 as a stem cell marker.** *J Histochem Cytochem* 2010, **58**:1093–1106.
5. Parte S, Bhartiya D, Telang J, Daithankar V, Salvi V, Zaveri K, Hinduja I : **Detection, characterization, and spontaneous differentiation in vitro of very small embryonic-like putative stem cells in adult mammalian ovary*.*** *Stem Cells Dev* 2011, **20**(8):1451-1464.
6. Mitsui K, Tokuzawa Y, Itoh H, Segawa K, Murakami M, Takahashi K, Maruyama M, Maeda M, Yamanaka S: **The homeoprotein Nanog is required for maintenance of pluripotency in mouse epiblast and ES cells.** *Cell* 2003, **113**(5):631-642.
7. Yamaguchi S, Kurimoto K, Yabuta Y, Sasaki H, Nakatsuji N, Saitou M, Tada T: **Conditional knockdown of Nanog induces apoptotic cell death in mouse migrating primordial germ cells.** *Development* 2009, **136**(23):4011-4020.
8. Jagarlamudi K, Rajkovic A: **Oogenesis: transcriptional regulators and mouse models.** *Mol Cell Endocrinol* 2012, 5:**356**(1-2):31-39.
9. Kehler J, Tolkunova E, Koschorz B, Pesce M, Gentile L, Boiani M, Lomelí H, Nagy A, McLaughlin KJ, Schöler HR, Tomilin A: [**Oct4 is required for primordial germ cell survival.**](http://www.ncbi.nlm.nih.gov/pubmed/15486564) *EMBO Rep* 2004, **5**(11):1078-1083.
10. Buehr M, McLaren A, Bartley A, and Darling S: **Proliferation and migration of primordial germ cells in We/We mouse embryos.** *Dev Dyn* 1993, **198**(3):182-189.
11. Yoshida H, Takakura N, Kataoka H, Kunisada T, Okamura H, Nishikawa SI: **Stepwise requirement of c-kit tyrosine kinase in mouse ovarian follicle development.** *Dev Biol* 1997, **184**(1):122-137.
12. Tanikawa M, Harada T, Mitsunari M, Onohara Y, Iwabe T, Terakawa N: **Expression of c-kit messenger ribonucleic acid in human oocyte and presence of soluble c-kit in follicular fluid.** *J Clin Endocrinol Metab* 1998, **83**(4):1239-1242.
13. Parrott JA, Skinner MK: **Kit-ligand/stem cell factor induces primordial follicle development and initiates folliculogenesis.** *Endocrinology* 1999, **140**(9):4262-4271.
14. Reynaud K, Cortvrindt R, Smitz J, Driancourt MA: **Effects of Kit Ligand and anti-Kit antibody on growth of cultured mouse preantral follicles.** *Mol Reprod Dev*. 2000, **56**(4):483-94.
15. Eppig JJ: **Oocyte control of ovarian follicular development and function in mammals.** *Reproduction* 2001, **122**(6):829-838.
16. Nilsson E, Skinner MK: **Cellular interactions that control primordial follicle development and folliculogenesis.** *J Soc Gynecol Investig* 2001, 8(1 Suppl Proceedings):S17-20.
17. Carlsson IB, Laitinen MP, Scott JE, Louhio H, Velentzis L, Tuuri T, Aaltonen J, Ritvos O, Winston RM, Hovatta O: **Kit ligand and c-Kit are expressed during early human ovarian follicular development and their interaction is required for the survival of follicles in long-term culture.** *Reproduction* 2006, **131**(4):641-9.
18. Hutt KJ, McLaughlin EA, Holland MK: **Kit ligand and c-Kit have diverse roles during mammalian oogenesis and folliculogenesis.** *Mol Hum Reprod* 2006, **12**(2):61-69.
19. Raz E: **The function and regulation of vasa-like genes in germ-cell development.** *Genome Biol* 2000, **1** REVIEWS 1017.
20. Tanaka SS, Toyooka Y, Akasu R, Katoh-Fukui Y, Nakahara Y, Suzuki R, Yokoyama M, Noce T: [**The mouse homolog of Drosophila Vasa is required for the development of male germ cells.**](http://www.ncbi.nlm.nih.gov/pubmed/10766740) *Genes Dev* 2000, **14**(7):841-853.
21. Castrillon DH, Quade BJ, Wang TY, Quigley C Crum CP: **The human VASA gene is specifically expressed in the germ cell lineage.** *Proc Natl Acad Sci USA* 2000, **97** 9585-9590.
22. Newmark PA, Wang Y, Chong T: **Germ cell specification and regeneration in planarians.** *Cold Spring Harb Symp Quant* *Biol* 2008, 73:573-581.
23. van Rooij IA, Broekmans FJ, te Velde ER, Fauser BC, Bancsi LF, de Jong FH, Themmen AP: [**Serum anti-Müllerian hormone levels: a novel measure of ovarian reserve.**](http://www.ncbi.nlm.nih.gov/pubmed/12456604) *Hum Reprod* 2002, **17**(12):3065-3071.
24. Schmidt KL, Kryger-Baggesen N, Byskov AG, Andersen CY: **Anti-Müllerian hormone initiates growth of human primordial follicles *in vitro.*** *Mol Cell Endocrinol* 2005, **234**(1-2):87-93.
25. Ebner T, Sommergruber M, Moser M, Shebl O, Schreier-Lechner E, Tews G: **Basal level of anti-Müllerian hormone is associated with oocyte quality in stimulated cycles.** *Hum Reprod* 2006, **21**(8):2022-2026.
26. Adhikari D, Liu K: **Molecular mechanisms underlying the activation of mammalian primordial follicles.** *Endocr Rev* 2009, **30**(5):438-464.
27. Dong J, Albertini DF, Nishimori K, Kumar TR, Lu N, Matzuk MM: **Growth differentiation factor-9 is required during early ovarian folliculogenesis.** *Nature* 1996, **383**(6600):531-535.
28. Vitt UA, McGee EA, Hayashi M, Hsueh AJ: [***In vivo* treatment with GDF-9 stimulates primordial and primary follicle progression and theca cell marker CYP17 in ovaries of immature rats.**](http://www.ncbi.nlm.nih.gov/pubmed/11014238) *Endocrinology* 2000, **141**(10):3814-3820.
29. Pangas SA, Choi Y, Ballow DJ, Zhao Y, Westphal H, Matzuk MM, Rajkovic A: [**Oogenesis requires germ cell-specific transcriptional regulators Sohlh1 and Lhx8.**](http://www.ncbi.nlm.nih.gov/pubmed/16690745) *Proc Natl Acad Sci U S A*  2006, **103**(21):8090-8095.
30. [Choi Y](http://www.ncbi.nlm.nih.gov/pubmed?term=Choi Y%5BAuthor%5D&cauthor=true&cauthor_uid=18509161), [Ballow DJ](http://www.ncbi.nlm.nih.gov/pubmed?term=Ballow DJ%5BAuthor%5D&cauthor=true&cauthor_uid=18509161), [Xin Y](http://www.ncbi.nlm.nih.gov/pubmed?term=Xin Y%5BAuthor%5D&cauthor=true&cauthor_uid=18509161), [Rajkovic A](http://www.ncbi.nlm.nih.gov/pubmed?term=Rajkovic A%5BAuthor%5D&cauthor=true&cauthor_uid=18509161). **Lim homeobox gene, lhx8, is essential for mouse oocyte differentiation and survival.** [*Biol Reprod*](http://www.ncbi.nlm.nih.gov/pubmed?term=Choi 2008 lhx8) 2008, **79**(3):442-449.
31. [Zhang LJ](http://www.ncbi.nlm.nih.gov/pubmed?term=Zhang LJ%5BAuthor%5D&cauthor=true&cauthor_uid=22796561), [Pan B](http://www.ncbi.nlm.nih.gov/pubmed?term=Pan B%5BAuthor%5D&cauthor=true&cauthor_uid=22796561), [Chen B](http://www.ncbi.nlm.nih.gov/pubmed?term=Chen B%5BAuthor%5D&cauthor=true&cauthor_uid=22796561), [Zhang XF](http://www.ncbi.nlm.nih.gov/pubmed?term=Zhang XF%5BAuthor%5D&cauthor=true&cauthor_uid=22796561), [Liang GJ](http://www.ncbi.nlm.nih.gov/pubmed?term=Liang GJ%5BAuthor%5D&cauthor=true&cauthor_uid=22796561), [Feng YN](http://www.ncbi.nlm.nih.gov/pubmed?term=Feng YN%5BAuthor%5D&cauthor=true&cauthor_uid=22796561), [Wang LQ](http://www.ncbi.nlm.nih.gov/pubmed?term=Wang LQ%5BAuthor%5D&cauthor=true&cauthor_uid=22796561), [Ma JM](http://www.ncbi.nlm.nih.gov/pubmed?term=Ma JM%5BAuthor%5D&cauthor=true&cauthor_uid=22796561), [Li L](http://www.ncbi.nlm.nih.gov/pubmed?term=Li L%5BAuthor%5D&cauthor=true&cauthor_uid=22796561), [Shen W](http://www.ncbi.nlm.nih.gov/pubmed?term=Shen W%5BAuthor%5D&cauthor=true&cauthor_uid=22796561): **Expression and epigenetic dynamics of transcription regulator Lhx8 during mouse oogenesis.** [*Gene*](http://www.ncbi.nlm.nih.gov/pubmed?term=Zhang 2012 lhx8) 2012, **506**(1):1-9.
32. [Shikone T](http://www.ncbi.nlm.nih.gov/pubmed?term=Shikone T%5BAuthor%5D&cauthor=true&cauthor_uid=1324147), [Yamoto M](http://www.ncbi.nlm.nih.gov/pubmed?term=Yamoto M%5BAuthor%5D&cauthor=true&cauthor_uid=1324147), [Nakano R](http://www.ncbi.nlm.nih.gov/pubmed?term=Nakano R%5BAuthor%5D&cauthor=true&cauthor_uid=1324147): **Follicle-stimulating hormone induces functional receptors for basic fibroblast growth factor in rat granulosa cells.** [*Endocrinology*](http://www.ncbi.nlm.nih.gov/pubmed?term=Shikone 1992%2C bFGF) 1992, **131**(3):1063-1068.
33. van Wezel IL, Umapathysivam K, Tilley WD, Rodgers RJ: **Immunohistochemical localization of basic fibroblast growth factor in bovine ovarian follicles.** *Mol Cell Endocrinol.* 1995, **115**(2):133-140.
34. [Wandji SA](http://www.ncbi.nlm.nih.gov/pubmed?term=Wandji SA%5BAuthor%5D&cauthor=true&cauthor_uid=16727844), [Eppig JJ](http://www.ncbi.nlm.nih.gov/pubmed?term=Eppig JJ%5BAuthor%5D&cauthor=true&cauthor_uid=16727844), [Fortune JE](http://www.ncbi.nlm.nih.gov/pubmed?term=Fortune JE%5BAuthor%5D&cauthor=true&cauthor_uid=16727844): **FSH and growth factors affect the growth and endocrine function in vitro of granulosa cells of bovine preantral follicles.** [*Theriogenology*](http://www.ncbi.nlm.nih.gov/pubmed?term=Wandji 1996%2C bFGF) 1996, **45**(4):817-832.
35. [Kezele P](http://www.ncbi.nlm.nih.gov/pubmed?term=Kezele P%5BAuthor%5D&cauthor=true&cauthor_uid=12161345), [Nilsson E](http://www.ncbi.nlm.nih.gov/pubmed?term=Nilsson E%5BAuthor%5D&cauthor=true&cauthor_uid=12161345), [Skinner MK](http://www.ncbi.nlm.nih.gov/pubmed?term=Skinner MK%5BAuthor%5D&cauthor=true&cauthor_uid=12161345): **Cell-cell interactions in primordial follicle assembly and development.** [*Front Biosci*](http://www.ncbi.nlm.nih.gov/pubmed/12161345) 2002, **7**:d1990-1996.
36. [Ben-Haroush A](http://www.ncbi.nlm.nih.gov/pubmed?term=Ben-Haroush A%5BAuthor%5D&cauthor=true&cauthor_uid=16210019), [Abir R](http://www.ncbi.nlm.nih.gov/pubmed?term=Abir R%5BAuthor%5D&cauthor=true&cauthor_uid=16210019), [Ao A](http://www.ncbi.nlm.nih.gov/pubmed?term=Ao A%5BAuthor%5D&cauthor=true&cauthor_uid=16210019), [Jin S](http://www.ncbi.nlm.nih.gov/pubmed?term=Jin S%5BAuthor%5D&cauthor=true&cauthor_uid=16210019), [Kessler-Icekson G](http://www.ncbi.nlm.nih.gov/pubmed?term=Kessler-Icekson G%5BAuthor%5D&cauthor=true&cauthor_uid=16210019), [Feldberg D](http://www.ncbi.nlm.nih.gov/pubmed?term=Feldberg D%5BAuthor%5D&cauthor=true&cauthor_uid=16210019), [Fisch B](http://www.ncbi.nlm.nih.gov/pubmed?term=Fisch B%5BAuthor%5D&cauthor=true&cauthor_uid=16210019): **Expression of basic fibroblast growth factor and its receptors in human ovarian follicles from adults and fetuses.** [*Fertil Steril*](http://www.ncbi.nlm.nih.gov/pubmed?term=Expression of basic fibroblast growth factor and its receptors in human ovarian follicles from adults and fetuses) 2005, (84 Suppl) **2**:1257-1268.
37. Nilsson E, Parrott JA, Skinner MK: [**Basic fibroblast growth factor induces primordial follicle development and initiates folliculogenesis.**](http://www.ncbi.nlm.nih.gov/pubmed/11325522) *Mol Cell Endocrinol* 2001,   **175**(1-2):123-130.
38. Nilsson EE, Skinner MK: [**Kit ligand and basic fibroblast growth factor interactions in the induction of ovarian primordial to primary follicle transition.**](http://www.ncbi.nlm.nih.gov/pubmed/15062541) *Mol Cell Endocrinol* 2004, **214**(1-2):19-25.
39. Nilsson E, Rogers N, Skinner MK: **Actions of anti-Mullerian hormone on the ovarian transcriptome to inhibit primordial to primary follicle transition.** *Reproduction* 2007, **134**(2):209-221.
40. Matos MH, Lima-Verde IB, Bruno JB, Lopes CA, Martins FS, Santos KD, Rocha RM, Silva JR, Báo SN, Figueiredo JR: **Follicle stimulating hormone and fibroblast growth factor-2 interact and promote goat primordial follicle development in vitro.** *Reprod Fertil Dev* 2007a, **19**(5):677-684.
41. Matos MH, van den Hurk R, Lima-Verde IB, Luque MC, Santos KD, Martins FS, Báo SN, Lucci CM, Figueiredo JR: [**Effects of fibroblast growth factor-2 on the in vitro culture of caprine preantral follicles.**](http://www.ncbi.nlm.nih.gov/pubmed/17536183) *Cells Tissues Organs* 2007b, **186**(2):112-120.
42. Garor R, Abir R, Erman A, Felz C, Nitke S, Fisch B: **Effect of basic fibroblast growth factor on in vitro development of human ovarian primordial follicles.** *Fertil Steril* 2009, **91**(5 Suppl):1967-1975.
43. Matos MH, Bruno JB, Rocha RM, Lima-Verde IB, Santos KD, Saraiva MV, Silva JR, Martins FS, Chaves RN, Báo SN, Figueiredo JR: ***In vitro* development of primordial follicles after long-term culture of goat ovarian tissue.** *Res Vet Sci* 2011, 90(3):404-411.
44. Aittomaki, K, Herva, R., Stenman, U.H. et al: **Clinical features of primary ovarian failure caused by a point mutation in the follicle-stimulating hormone receptor gene.** *J. Clin. Endocrinol. Metab* 1996 **81**, 3722–3726.
45. O'Shaughnessy PJ, Dudley K, Rajapaksha WR: **Expression of follicle stimulating hormone-receptor mRNA during gonadal development.** *Mol Cell Endocrinol* 1996, **125**(1-2):169-175.
46. [Joyce IM](http://www.ncbi.nlm.nih.gov/pubmed?term=Joyce IM%5BAuthor%5D&cauthor=true&cauthor_uid=10525339), [Pendola FL](http://www.ncbi.nlm.nih.gov/pubmed?term=Pendola FL%5BAuthor%5D&cauthor=true&cauthor_uid=10525339), [Wigglesworth K](http://www.ncbi.nlm.nih.gov/pubmed?term=Wigglesworth K%5BAuthor%5D&cauthor=true&cauthor_uid=10525339), [Eppig JJ](http://www.ncbi.nlm.nih.gov/pubmed?term=Eppig JJ%5BAuthor%5D&cauthor=true&cauthor_uid=10525339): **Oocyte regulation of kit ligand expression in mouse ovarian follicles.** [*Dev Biol*](http://www.ncbi.nlm.nih.gov/pubmed?term=Joyce 1999%2C FSH) 1999, **214**(2):342-53.
47. Burns KH, Yan C, Kumar TR, Matzuk MM: [**Analysis of ovarian gene expression in follicle-stimulating hormone beta knockout mice.**](http://www.ncbi.nlm.nih.gov/pubmed/11415992) *Endocrinology* 2001, **142**(7):2742-2751.
48. Méduri G, Charnaux N, Driancourt MA, Combettes L, Granet P, Vannier B, Loosfelt H, Milgrom E: **Follicle-stimulating hormone receptors in oocytes?** *J Clin Endocrinol Metab* 2002, **87**(5):2266-2276.
49. Abel MH, Huhtaniemi I, Pakarinen P, Kumar TR, Charlton HM: [**Age-related uterine and ovarian hypertrophy in FSH receptor knockout and FSH beta subunit knockout mice.**](http://www.ncbi.nlm.nih.gov/pubmed/12578530) *Reproduction* 2003, **125**(2):165-173.
50. Thomas FH, Ethier JF, Shimasaki S, Vanderhyden BC: [**Follicle-stimulating hormone regulates oocyte growth by modulation of expression of oocyte and granulosa cell factors.**](http://www.ncbi.nlm.nih.gov/pubmed/15539559) *Endocrinology* 2005, **146**(2):941-949.
51. [van den Hurk R](http://www.ncbi.nlm.nih.gov/pubmed?term=van den Hurk R%5BAuthor%5D&cauthor=true&cauthor_uid=15763114), [Zhao J](http://www.ncbi.nlm.nih.gov/pubmed?term=Zhao J%5BAuthor%5D&cauthor=true&cauthor_uid=15763114): **Formation of mammalian oocytes and their growth differentiation and maturation within ovarian follicles.** [*Theriogenology*](http://www.ncbi.nlm.nih.gov/pubmed/15763114) 2005, **63**(6):1717-1751.
52. Matos MH, Lima-Verde IB, Luque MC, Maia JE Jr, Silva JR, Celestino JJ, Martins FS, Báo SN, Lucci CM, Figueiredo JR: [**Essential role of follicle stimulating hormo ne in the maintenance of caprine preantral follicle viability *in vitro*.**](http://www.ncbi.nlm.nih.gov/pubmed/17462110) *Zygote* 2007, **15**(2):173-182.
53. Bhartiya D, Sriraman K, Gunjal P, Modak H: **Gonadotropin treatment augments postnatal oogenesis and primordial follicle assembly in adult mouse ovaries?** *J Ovarian Res* 2012b, **5**(1):32.
